# Development of cavitary lung disease as a long-term complication of coronavirus disease 2019 in a young previously healthy patient: a case report

**DOI:** 10.1186/s13256-021-02961-9

**Published:** 2021-07-13

**Authors:** Goar Egoryan, Elise Hyser, Ammar H. Mushtaq, Maria Adriana Yanez-Bello, Daniela Patricia Trelles-Garcia, Harvey J. Friedman, Guillermo Rodriguez-Nava

**Affiliations:** 1 Department of Internal Medicine, AMITA Health Saint Francis Hospital, 355 Ridge Ave, Evanston, IL 60202 USA; 2 Critical Care Units, AMITA Health Saint Francis Hospital, Evanston, IL USA; 3 Clinical Associate Professor of Medicine, University of Illinois College of Medicine, Chicago, IL USA

**Keywords:** SARS-CoV-2, COVID-19, Chest computed tomography, Cavitary lung lesion

## Abstract

**Background:**

Cavities are frequent manifestations of a wide variety of pathological processes involving the lung. There has been a growing body of evidence of coronavirus disease 2019 leading to a cavitary pulmonary disease.

**Case presentation:**

A healthy 29-year-old Filipino male presented to the hospital a couple of months after convalescence from coronavirus disease 2019 with severe pleuritic chest pain, fever, chills, and shortness of breath, and was found to have a cavitary lung lesion on chest computed tomography. While conservative management alone failed to improve the patient’s condition, he ultimately underwent left lung video-assisted thoracoscopic surgery decortication. Even though the surgical pathology revealed only necrosis with dense acute inflammation and granulation tissue with no microorganisms, he gradually improved with medical therapy adjunct with surgical therapy.

**Conclusion:**

Documented cases of cavitary lung disease secondary to coronavirus disease 2019 have been mostly reported in the acute or subacute phase of the infection. However, clinicians should recognize this entity as a late complication of coronavirus disease 2019, even in previously healthy individuals.

## Background

Cavitary lung disease may result from several pathological processes, including suppurative necrosis, caseous necrosis, ischemic necrosis, displacement of lung tissue by cystic structures, and cystic dilatation of lung structures, vasculitis, or high-pressure traumas [1, 2]. No single factor differentiates organisms frequently associated with pulmonary cavitation from organisms rarely associated with pulmonary cavitation. In general, organisms that cause subacute or chronic lung infections (for example, mycobacteria and fungi) are more frequently associated with cavitary lung disease than organisms that cause acute lung infections (for example, viruses and pneumococcus) [1]. However, cavitary lung disease has been documented as a complication of acute viral infections, including severe acute respiratory virus (SARS) and Middle East respiratory virus (MERS) infections [1, 3, 4].

After its identification in December 2019, the novel severe acute respiratory syndrome coronavirus 2 (SARS-CoV-2) has caused a sudden significant increase in hospitalizations for pneumonia with multiorgan disease with acute complications that include impaired function of the heart, brain, lung, liver, kidney, and coagulation system [5]. Long-term outcomes from the coronavirus disease 2019 (COVID-19) are currently unknown, but recent data have emerged that some patients continue to experience symptoms and complications related to COVID-19 after the acute infection, the so-called post-acute COVID-19 syndrome [5, 6]. Lung cavitation is an uncommon complication and has been mostly reported in the acute and subacute phases of the infection [3, 7–11]. Here we report a previously healthy young patient who developed cavitary lung disease 6 months after first onset of symptoms.

## Case presentation

A previously healthy 29-year-old Filipino male presented with pleuritic chest pain, exertional dyspnea, fever, chills, and a 9-kg weight loss over the past 3 months. His medical history was only remarkable for mild COVID-19 with a few days of febrile illness and loss of sense of taste and smell in March 2020. He was not hospitalized and did not undergo any imaging at that time. He was a resident in the Chicago metropolitan area, lived with a male partner, and had a dog. He described his apartment as very humid, where he had several plants, some of which were growing mushrooms in the plant pots. He worked as a designer from home, although disclosed traveling to California, Indiana, and Wisconsin in the past 3 weeks before presentation, but denied any outdoor activities. He had no relevant family or surgical history, denied known allergies to foods or medications, and had no history of sexually transmitted diseases.

**Fig. 1:**
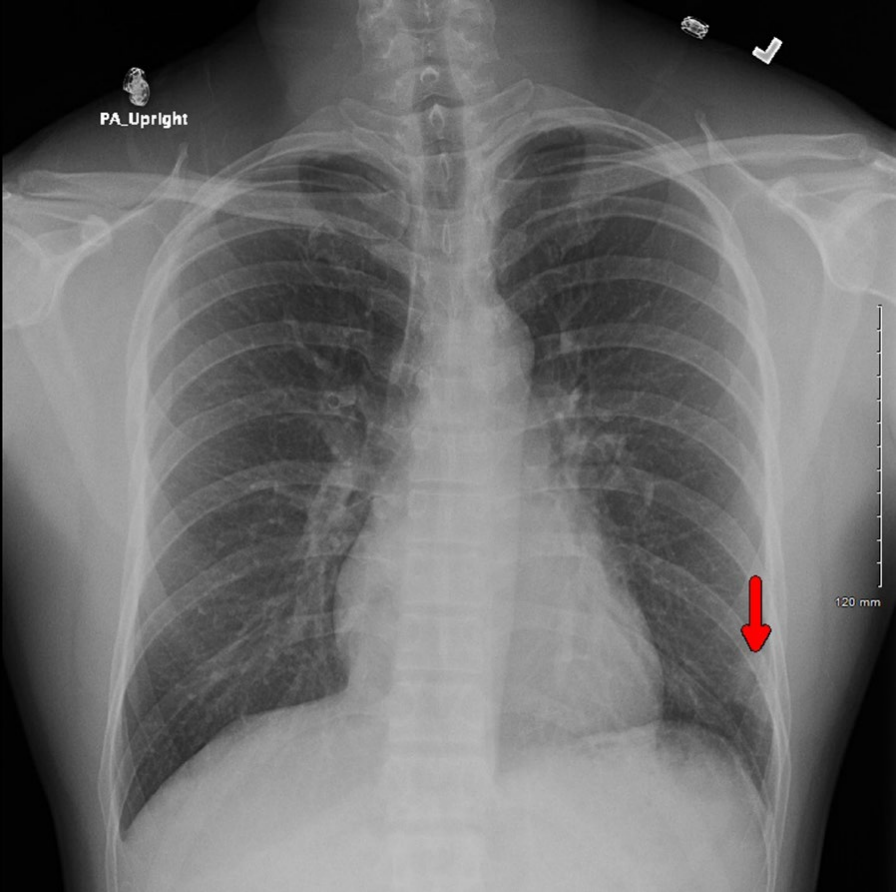
Chest radiography showing a lobular irregularly shaped soft-tissue density at the left base laterally (red arrow)

**Fig. 2:**
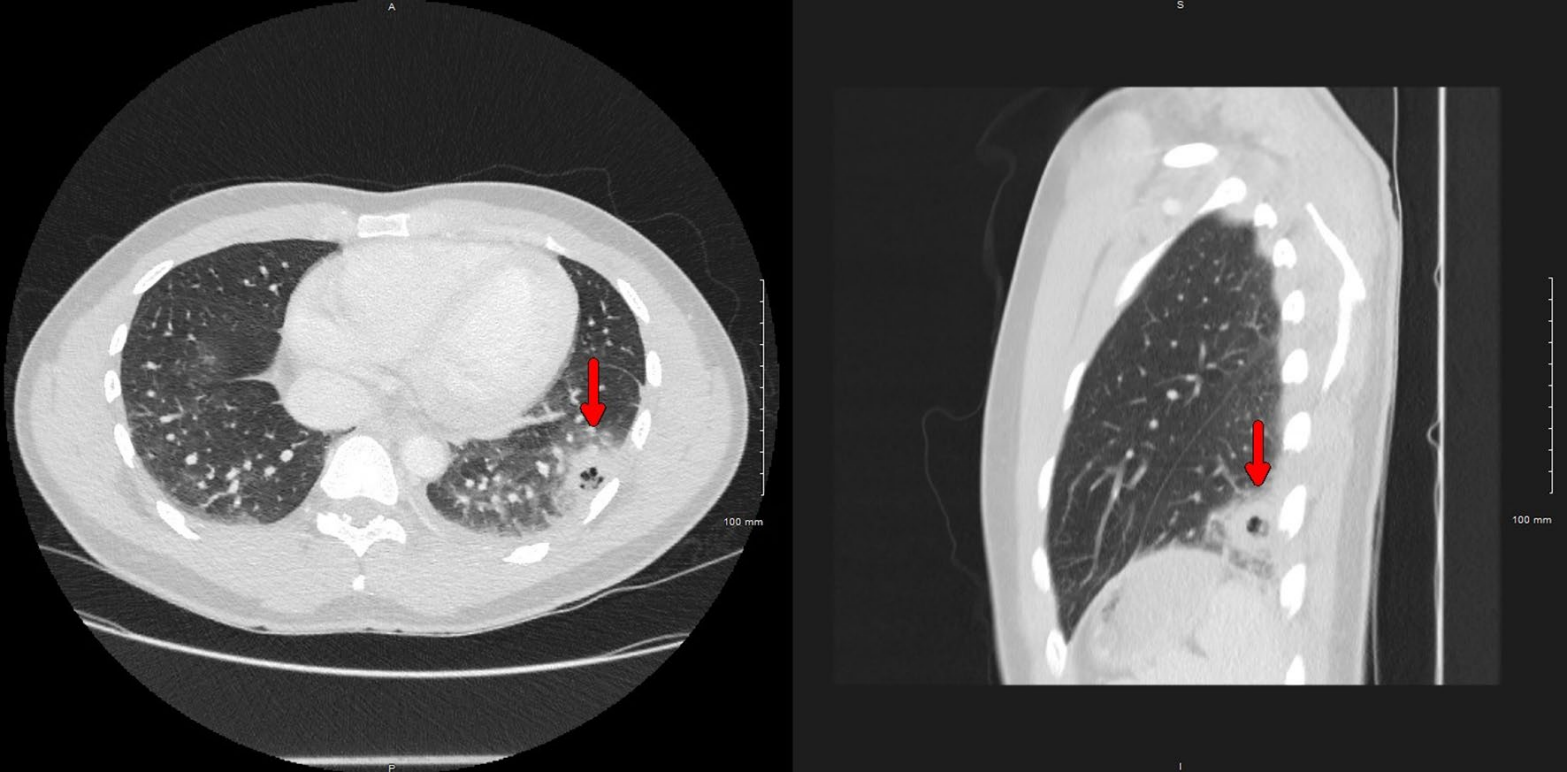
Chest CT showing a left lower lobe cavitating mass measuring up to 3 cm (red arrows) and peripheral ground-glass opacities within the lung bases bilaterally (right: axial plane; left: sagittal plane)

His vital signs were notable for fever up to 38.6 °C, heart rate of 101 beats/minute, respiratory rate of 20 breaths/minute, blood pressure of 120/60 mmHg, and saturation of 98% on ambient air. He was in distress secondary to intense chest pain, and the physical exam was only remarkable for a rash in the right cubital fossa. Initial laboratory testing showed leukocytosis (14.1 × 10^9^/L, reference 4.0–11 × 10^9^/L), with neutrophilia (11.3 × 10^9^/L, reference 1–7 × 10^9^/L) and monocytosis (1.2 × 10^9^/L, reference 0.3–1.0 × 10^9^/L), and abnormal C-reactive protein levels (14.5 mg/dL, reference < 1.0 mg/dL). Chest x-ray demonstrated a lobular irregularly shaped soft-tissue density at the left base laterally (Fig. 1). Computed tomography (CT) with contrast of the chest showed a left lower lobe 3-cm cavitating mass and peripheral ground-glass opacities within the lung bases bilaterally (Fig. 2). The patient was admitted to the general medical floor, and empirical therapy with broad-spectrum antibiotics—intravenous vancomycin and piperacillin–tazobactam—was started. Multiple tests were sent to assess the etiology of the cavitary lesion, including a rapid human immunodeficiency virus (HIV) 1/2 test, *Blastomyces dermatitidis* and *Histoplasma capsulatum* urine antigens, *Coccidioides immitis* antibodies, *Cryptococcus neoformans* serum antigen, *Aspergillus* antibodies, interferon-gamma release assay for latent tuberculosis, SARS-CoV-2 Reverse-transcription polymerase chain reaction (RT-PCR), PCR for the detection of methicillin-resistant *Staphylococcus aureus*, and blood cultures, all of which were reported negative.

On hospital day 3, a newly developed left-sided pleural effusion was observed on chest x-rays. Antifungal therapy with itraconazole was added, and diagnostic thoracentesis was performed with successful drainage of 140 mL of pleural fluid. The fluid analysis showed an exudative effusion pattern with a pH of 8.5, 498 × 10^9^/L white blood cells, glucose of 61 mg/dL, fluid protein of 3.8 g/dL (serum protein 6.3 g/L, pleural-fluid-to-serum ratio 0.6), and lactate dehydrogenase (LDH) of 1332 U/L (serum LDH 192 U/L, pleural-fluid-to-serum ratio 6.9), but subsequent acid-fast smear, blood, and fungal cultures came back negative. The patient started to improve, and antibiotic treatment was deescalated to cefdinir with the addition of itraconazole given epidemiological risk factors for dimorphic fungal infections (Midwest resident and recent travel to California). He was ultimately discharged on oral therapy (fluconazole, trimethoprim–sulfamethoxazole, and cefdinir) after 5 days with significant clinical improvement.

One week after discharge, the patient returned with constitutional symptoms, pleuritic pain, and cough productive of foul-smelling greenish-yellow sputum that tasted like sulfur. Chest CT showed a new left basilar empyema with air and fluid present within this collection and consolidation in the lingula surrounding the empyema. During this hospitalization, he was tested for antinuclear antibodies (ANA) and anti-neutrophil cytoplasmic antibodies (P-ANCA and C-ANCA) to rule out an autoimmune disease with negative results. The antimicrobial regimen at that point included intravenous meropenem, vancomycin, and voriconazole.

He finally underwent bronchoscopy and left video-assisted thoracoscopic surgery (VATS) decortication with chest tube placement postoperatively and was started on antibiotics with anaerobic coverage. Surgical pathology revealed necrosis with dense acute inflammation and granulation tissue but no microorganisms. Throughout the hospitalization, breathing and cough improved, he remained afebrile, and leukocytosis resolved. The chest tubes were removed after 3 days, and he was subsequently discharged on oral amoxicillin–clavulanate 875–125 mg every 12 hours for 30 days. Despite all the negative cultures, which was expected given the protracted course of antimicrobial therapy, it was concluded that the patient most likely experienced necrotizing bacterial lung infection. Since cavitary lung disease is uncommon in an otherwise healthy young adult, and we ruled out all other conditions from the list of differential diagnoses, it was deduced that a sequela complication of COVID-19 was most likely.

One month after discharge, the patient reported his condition to be progressively improving, but he still reported mild pleuritic pain with deep inspirations and stated that his exercise capacity decreased drastically. Chest x-rays showed improvement in lung lesions.

## Discussion and conclusion

We present a young, healthy adult who developed cavitary lung disease 6 months after mild COVID-19 illness. To date, documented cases of cavitary lung disease secondary to COVID-19 have been reported in the acute or subacute phase of the infection. A 27-year-old man hospitalized with COVID-19 was found to have cavitation in the apical segment of the lower lobe of the lung [8], and a case series described 12 patients with severe COVID-19 that developed lung cavitation during the acute phase of the illness [11]. One case reported a 37-year-old man who developed multiple small lung cavities 2 weeks after convalescence from COVID-19 [10], while another case report described a 34-year-old male diagnosed with a large lung cavity 2 weeks after being discharged from the hospital [3]. Furthermore, a 52-year-old male developed a lung cavity 3 weeks after the diagnosis of COVID-19 was made [7]. Lastly, two 59-year-old men were reported to develop spontaneous large pulmonary cavities 1 month after COVID-19 [9]. Hence, clinicians should be aware of cavitary lung disease as an acute and late complication of COVID-19.

Cavitation is one of the least frequent complications in COVID-19, and appropriate differential diagnosis workup should be done before tying cavity development to COVID-19 [12, 13]. Other processes, such as bacterial superinfection or coinfection with *Mycobacterium tuberculosis*, should be ruled out [14–16]. In our case, an extensive workup was performed, and given the fact that our patient had no previous significant comorbidities, it was concluded that his cavitary lung disease was a long-term complication of COVID-19.

The cavitation mechanism in COVID-19 is unknown and may result from intense inflammatory response leading to diffuse alveolar damage, intraalveolar hemorrhage, and necrosis of parenchymal cells [17, 18]. It is worth mentioning that our patient had just a mild course of COVID-19, and nevertheless, he developed a significant lung sequela. Further studies are needed to understand the exact pathophysiologic mechanism underlying cavitary lung disease as an acute and late complication of COVID-19.

To date, there is no consensus on how post-COVID-19 cavities should be managed. Most of the few reported cases have been managed medically, with only one case reporting excision of infected pneumatoceles [19]. Our patient failed medical therapy alone and was required to undergo surgery combined with prolonged antibiotic therapy, highlighting the importance of a multidisciplinary approach in patients with COVID-19.

In conclusion, we present the case of a young adult who developed cavitary lung disease 6 months after a mild COVID-19 illness who failed medical therapy alone but was later successfully treated with combined surgical and medical therapy. Hopefully, as the pandemic unfolds, the whole range of long-term health effects associated with COVID-19 will be elucidated, and appropriate treatment guidelines for managing these complications, including cavitary lung disease, will be developed.

## Data Availability

The data used to support the findings of this study are available from the corresponding author on request, except for the patient’s personal health information owing to Health Insurance Portability and Accountability regulations.

## Abbreviations

ANA: Antinuclear antibodies
C-ANCA: Anti-neutrophil cytoplasmic antibodies
CT: Computed tomography
COVID-19: Coronavirus disease 2019
MERS: Middle East respiratory virus
P-ANCA: Perinuclear anti-neutrophil cytoplasmic antibodies
RT-PCR: Reverse-transcription polymerase chain reaction
SARS: Severe acute respiratory syndrome
SARS-CoV-2: Severe acute respiratory syndrome coronavirus 2
VATS: Video-assisted thoracoscopic surgery
